# A New Kinect V2-Based Method for Visual Recognition and Grasping of a Yarn-Bobbin-Handling Robot

**DOI:** 10.3390/mi13060886

**Published:** 2022-05-31

**Authors:** Jinghai Han, Bo Liu, Yongle Jia, Shoufeng Jin, Maciej Sulowicz, Adam Glowacz, Grzegorz Królczyk, Zhixiong Li

**Affiliations:** 1 Institute of Rail Transport, Nanjing Vocational Institute of Transport Technology, Nanjing 211188, China; hanjinghai@njitt.edu.cn; 2 College of Mechanical and Electrical Engineering, Xi’an Polytechnic University, Xi’an 710600, China; 200221059@stu.xpu.edu.cn (B.L.); jiayle@163.com (Y.J.); jdxyjsf@126.com (S.J.); 3 Department of Electrical Engineering, Cracow University of Technology, 31-155 Cracow, Poland; maciej.sulowicz@pk.edu.pl (M.S.); adglow@agh.edu.pl (A.G.); 4 Department of Manufacturing Engineering and Automation Products, Opole University of Technology, 45-758 Opole, Poland; g.krolczyk@po.opole.pl; 5 Yonsei Frontier Lab, Yonsei University, Seoul 03722, Korea

**Keywords:** machine vision, industrial robots, yarn bobbin identification, robot control

## Abstract

This work proposes a Kinect V2-based visual method to solve the human dependence on the yarn bobbin robot in the grabbing operation. In this new method, a Kinect V2 camera is used to produce three-dimensional (3D) yarn-bobbin point cloud data for the robot in a work scenario. After removing the noise point cloud through a proper filtering process, the M-estimator sample consensus (MSAC) algorithm is employed to find the fitting plane of the 3D cloud data; then, the principal component analysis (PCA) is adopted to roughly register the template point cloud and the yarn-bobbin point cloud to define the initial position of the yarn bobbin. Lastly, the iterative closest point (ICP) algorithm is used to achieve precise registration of the 3D cloud data to determine the precise pose of the yarn bobbin. To evaluate the performance of the proposed method, an experimental platform is developed to validate the grabbing operation of the yarn bobbin robot in different scenarios. The analysis results show that the average working time of the robot system is within 10 s, and the grasping success rate is above 80%, which meets the industrial production requirements.

## 1. Introduction

Textile production, as an important resource for fabrics, clothing, and towels, is a typically labor-intensive industry [1]. Winding is a key process in textile production, which generally warps the yarn in the bobbins [2]. Currently, the yarn bobbins are often made by human workers. In order to reduce inefficient labor demands and costs, the textile industry is embracing AI (artificial intelligence) by using unmanned robots to complete the yarn bobbin production [3].

Current research in industrial robots focuses on robot grabbing with machine vision and graphics processing. Xue et al. [4] proposed a vision-based joint inspection method to improve the tracking accuracy of the weld robots. Ramon-Soria et al. [5] mounted a robotic arm with a camera on top of an unmanned aerial vehicle to achieve the grasping of aerial targets. D’Avella et al. [6] addressed the problem of grasping in a cluttered environment by using vision techniques to extract the edge contours of the target to calculate the grasping point and solve the perception problem caused by the chaotic environment. Jiang et al. [7] used information fusion of color images and depth images to improve the detection success rate of a vision robot. Du et al. [8] proposed a binocular vision-based object recognition and robotic grasping strategy. Yang et al. [9] built a vision recognition system (VRS) based on the YOLOv3 model to improve the efficiency of picking robots. Matsuo et al. [10] investigated the robots in logistics handling in warehouses and convenience stores, where multi-object grasping was achieved through the dual-arm operation. Xiao et al. [11] designed an automatic sorting robot that used depth information and infrared images to locate objects. Lin et al. [12] proposed a method to solve the occlusion problem by using multiple cameras to capture images and successfully grasp objects in the occluded area. Gao et al. [13] developed a vision-based seal assembly system to improve the efficiency of production lines. Sun et al. [14] proposed an end-to-end deep neural network-based grasping system that successfully grasped targets in the presence of object overlap. Song et al. [15] proposed a tactile-visual fusion-based robot grasping detection method to improve the applicability of manipulators in force-sensitive tasks. Yu et al. [16] proposed a new vision-based grasping method for target objects in occlusion situations. Bergamini et al. [17] proposed a method based on deep learning techniques for detecting the grasp of unknown objects by a robot in an unstructured environment. Hu et al. [18] established a projection mapping relationship between plane and space and proposed a 6D pipeline pose estimation method based on machine vision. Han et al. [19] applied binocular vision to identify and locate targets and improved the accuracy of robotic arm grasping. Lu et al. [20] applied machine vision to a sorting robot and developed a method that combined the YOLOv3 algorithm with manual features to improve sorting efficiency. Lou et al. [21] proposed a Gaussian hybrid robotic arm recognition and grasping method based on machine vision models to improve the grasping accuracy of the robot. According to the existing literature, most robot grabbing technologies address the multidimensional spatial position recognition of a single object and focus on the pose recognition of multiple objects with similar shape parameters; however, very limited work has addressed the challenging task of grabbing multiple objects with overlapping poses. As a result, most existing methods cannot be directly used in yarn bobbin robots; and to our best knowledge, little research has been done on grabbing multiple yarn bobbins with overlapping poses.

To bridge the aforementioned research gap, this work develops a new machine vision method to realize effective robot grabbing of multiple yarn bobbins. This new method is able to effectively recognize the spatial positions and poses of the yarn bobbins in cylindrical/conical shapes. This is the first time that a visual system has been developed for the robots in the practical textile application as a solution for yarn-bobbin grabbing. The visual system can obtain the target imaging and point cloud in specific scenarios with the Kinect V2 camera, derive precise 3D information of the yarn bobbins through target recognition and point cloud processing, and enable the industrial robots to correctly grab the yarn bobbins. 

This paper is structured as follows. In Section 2, the proposed machine vision method is introduced in detail. In Section 3, the experimental testing method is described and evaluated. Section 4 presents the main conclusions of this study.

## 2. Method and Materials

### 2.1. Establishment of Experimental Platform

**Figure 1:**
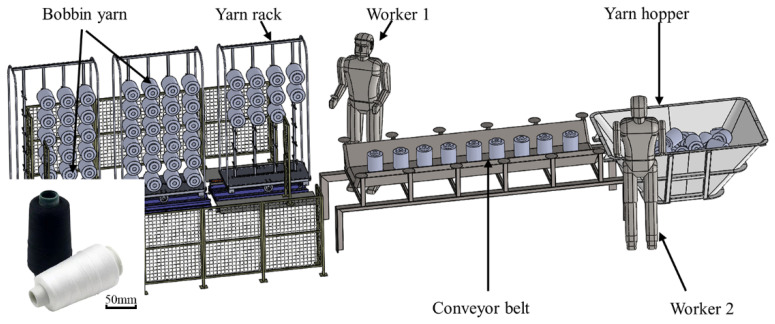
Manual yarn feeding process.

**Figure 2:**
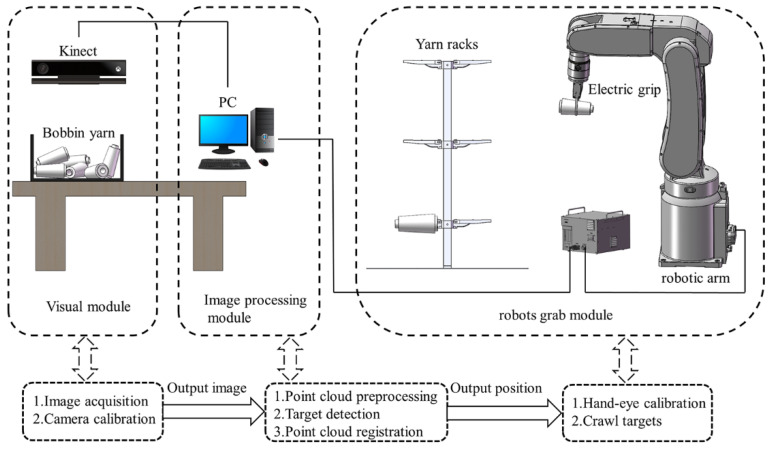
Developed a robot-based winding system.

In the textile winding process, the yarn bobbins produced by the winder machines will be collected and sorted into the yarn hopper, as shown in Figure 1, where one human laborer (worker 1) is employed to put the yarn bobbins from the hopper to the conveyor belt, and another human laborer (worker 2) is employed to hang the yarn bobbins onto the rack. As a result, at least two workers are required in the winding process to transfer the yarn bobbins from the hopper to the rack. Several repetitive operations of the workers will significantly increase labor intensity and safety risks. With the rapid development of AI (artificial intelligence) robotics, the labor-to-robot transition is a must in the textile winding process demanded by the textile industry; and at the first step in the roboticization reformation, intelligent robots are expected to handle the yarn bobbin to the rack to minimize manpower, increase efficiency and ensure staff safety. In this study, we aim to develop an industrial robot that can directly grab the yarn bobbins from the hopper and place them onto the rack. Compared with manual handling, this new robot-based winding system simples the operation complexity by removing the conveyor belt part. Figure 2 manifests the new winding system.

In Figure 2, the hardware of the new robot-based winding system consists of one Kinect V2 camera, one HIWIN RA605 robotic arm, one HIWIN XEG-32 electric gripper, and one computer. The computer connects with the Kinect V2 camera through a USB 3.0 interface and the robot arm control cabinet through Ethernet; the robot arm control cabinet controls the opening or closing of the electric gripper through I/O signals to realize the yarn-bobbin-grabbing function. The MATLAB programming language is adopted to develop each module in the system to enable the robots with visual recognition ability to effectively complete the grasping operation. According to the functional requirements of each module, the software system is divided into three parts: the vision module, the image processing module, and the robot grab module.

### 2.2. Vision Image Processing

**Figure 3:**
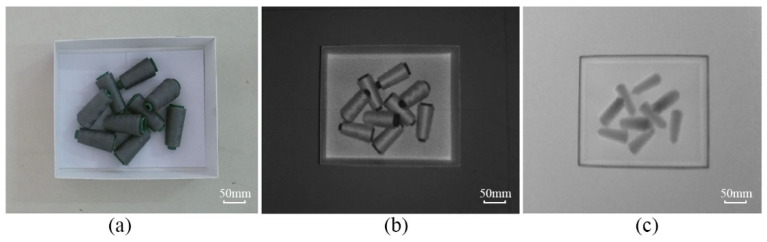
Kinect V2 acquired images: (**a**) RGB image; (**b**) IR image; (**c**) Depth image.

The Kinect V2 camera is used in the vision system to collect the images of the yarn bobbins. This device is based on the Kinect for Windows SDK, a development tool provided by Microsoft, to acquire the images of the yarn bobbins, as shown in Figure 3.

**Figure 4:**
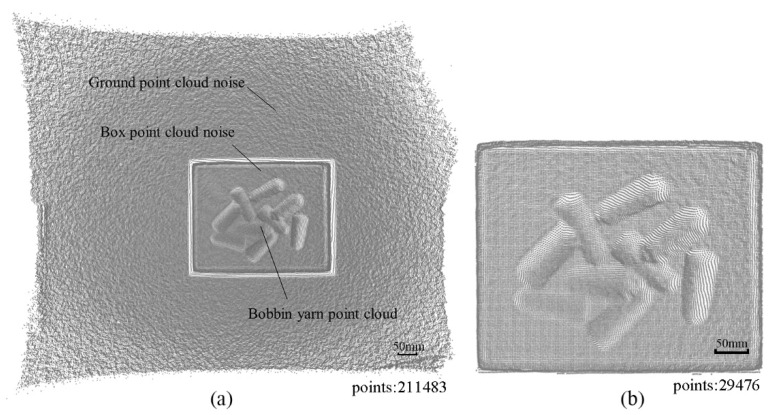
Mapping results: (**a**) point cloud of yarn bobbin; (**b**) denoised point cloud.

In Figure 3c, the depth image is often expressed in grayscale or pseudo-color, which is essentially different from the grayscale and RGB images of traditional cameras. The value corresponding to each pixel in the ordinary color image is the component of the pixel in the three RGB channels, and the value corresponding to each pixel in the depth image represents the spatial distance from the object surface point to the sensor plane. Therefore, the depth image can be converted into 3D point cloud data through coordinate transformation according to the characteristics of the depth image. The transformed point cloud data is shown in Figure 4a.

In Figure 4a, the obtained yarn bobbin point cloud data contains a lot of noise, and the data storage space is huge. Therefore, it is requested to filter the point cloud [22]. Pass-through filtering is employed as the filtering algorithm. By setting the threshold parameter, the points within the specified parameter range can be retained, while the outside points are filtered out. The detailed filtering process is described in the following steps:

(1) Import the point cloud data into the pass-through filter.

(2) Set different thresholds to determine the filtering directions.

(3) Filter the discrete points and save the denoised point cloud data.

The value ranges of the filter specified in this paper in *X*, *Y* and *Z* dimensions are set as follows:

(1) $$\{\begin{array}{c} -0.3\leq X<0.15\\ -0.15\leq Y<0.23\\ -1\leq Z<1 \end{array}$$

According to the dimensional values set in Equation (1), the original point cloud data shown in Figure 4a is straightly filtered to remove the redundant environmental point cloud, and the result is shown in Figure 4b.

**Figure 5:**
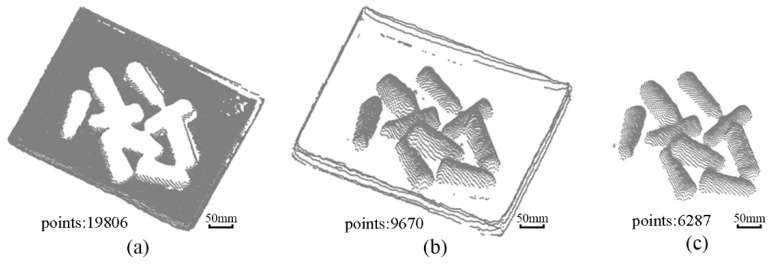
Extract yarn bobbin images via MSAC: (**a**) fitting the box bottom point cloud; (**b**) removing the box bottom point cloud; (**c**) removing the box side point cloud.

Then, the M-estimated sampling consensus algorithm (MSAC) [23] is adopted to fit the denoised point cloud to a bottom surface box, as shown in Figure 5a. The box side point cloud data is then removed by the direct-pass filtering to separate yarn bobbins from the box, as shown in Figure 5b,c.

### 2.3. Identification of Yarn Bobbin Targets

In order to identify each yarn bobbin in Figure 5c, the first step is to extract the key points. The key points are those with representative features such as stability and distinctiveness in the image. In this study, the corner points, gradients and grayscale sharply changing points in the point cloud are used as key points. The Internal Shape Descriptor (ISS) [24] is employed to use rich geometric information to extract the key points. Let the point $P_{i}(x_{i},y_{i},z_{i})$ be a point in the point cloud of one yarn bobbin; the process of extracting the key points is described as follows.

(1) Establish a local coordinate system for point *P_i_* in the point cloud of the yarn bobbin and set the search radius *r* for the point.

(2) Set point *P_i_* as the center and *r* as the neighbourhood radius, traverse all points within the neighbourhood of *P_i_,* and calculate their weights *w_ij_*.

(2) $$w_{ij}=1/||P_{i}-P_{j}\|,\;\left|P_{i}-P_{j}\right|<r$$

(3) Calculate the covariance matrix cov(*P_i_*) for each point *P_i_*.

(3) $$\mathrm{cov}\left(P_{i}\right)=\sum_{\left|P_{i}-P_{j}\right|<r}w_{ij}\left(P_{i}-P_{j}\right){\left(P_{i}-P_{j}\right)}^{T}/\sum_{\left|P_{i}-P_{j}\right|<r}w_{ij}$$

(4) Calculate the eigenvalues $\left\{\lambda_{i}^{1},\lambda_{i}^{2},\lambda_{i}^{3}\right\}$ of the covariance matrix of each point *P_i_* and rank them in descending order.

(5) Set the threshold (*ε*_1_ ≤ 1 and *ε*_2_ ≤ 1) and use the points satisfying Equation (4) as the key points of the yarn target.

(4) $$\lambda_{i}^{2}/\lambda_{i}^{1}\leq \varepsilon_{1},\;\lambda_{i}^{3}/\lambda_{i}^{2}\leq \varepsilon_{2}$$

(6) Repeat steps (1) to (5) until all the points in the yarn bobbin point cloud are traversed.

(7) Downsample the final extracted target key points.

**Figure 6:**
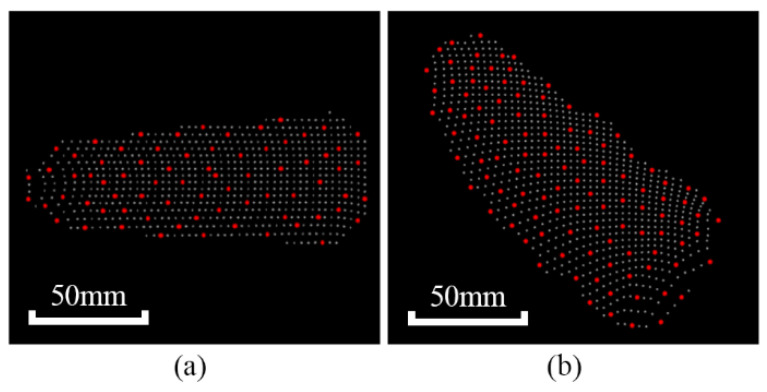
Key point extraction: (**a**) extraction result of the point cloud of the yarn bobbin template; (**b**) extraction result of the point cloud of the yarn bobbin to be captured.

Figure 6 provides an example of the key point extraction.

**Figure 7:**
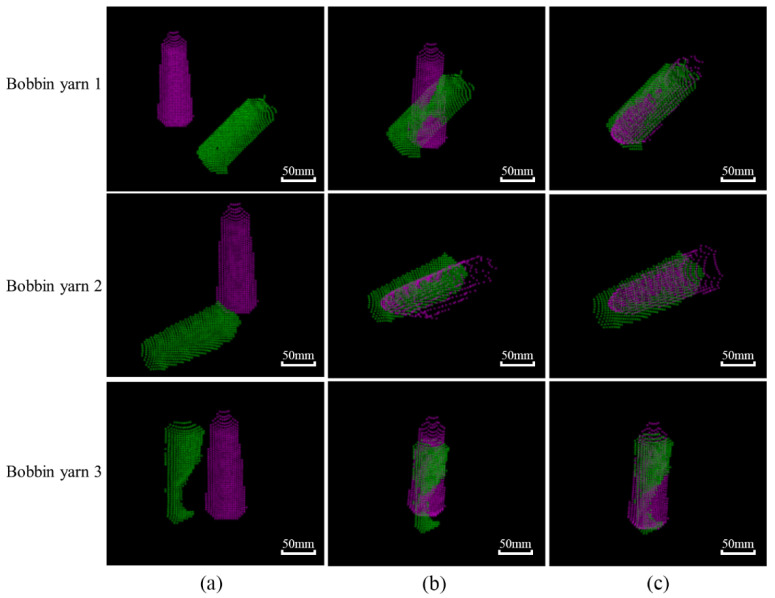
Point cloud alignment results: (**a**) initial position; (**b**) coarse alignment; (**c**) fine alignment.

After generating the key points, the second step is to perform the point cloud alignment between the template and target point cloud. Point cloud registration is to use the yarn bobbin template point cloud to construct point-to-point features to find the yarn bobbin target in the actual scene and determine its 3D pose. The rigid transformation between the template point cloud and the target point cloud is the estimation process of target poses. The registration effects are shown in Figure 7.

The rough registration based on the Principal Component Analysis (PCA) [25] is introduced in the point cloud alignment. The principal axis direction of the point cloud data is calculated for the rough registration, where the rotation matrix and the translation vector in the direction of the principal axis are derived by calculating the offset of the center coordinates of two sets of point clouds. Through the rough registration of point clouds, the target point cloud aligns the template point cloud with relatively low accuracy; it is necessary to minimize the error between the two-point clouds through continuous iteration, i.e., the precise registration. The ICP algorithm, also known as the iterative nearest-point algorithm, is widely used in the fine alignment phase of 3D point clouds because of its fast and simple nature [26]. This paper uses the point-to-point ICP algorithm for fine alignment of point clouds.

The core equation for the point-to-point ICP alignment is given in Equation (5), and the positional matrix for the yarn bobbin 1 in Figure 7 is described in Equation (6).

(5) $$\Delta T=\underset{R,t}{\arg\min}\sum_{i=1}^{n}{\left\|p_{bi}-\left(Rp_{ai^{\prime }}+t\right)\right\|}_{2}^{2}$$

(6) $$\left[\begin{array}{cccc} 0.76802416 & -0.62644737 & 0.13305101 & -0.05595922\\ 0.61897762 & 0.77942243 & 0.09678518 & 0.05432455\\ -0.16433376 & 0.00802224 & 0.98637217 & 0.01234947\\ 0 & 0 & 0 & 1.0000 \end{array}\right]$$

## 3. Numerical and Experimental Analyses

### 3.1. Calibration Experiments

The experimental platform is shown in Figure 2. The Kinect V2 camera was mounted on the upper part of the load table through the gantry and camera head device and could be adjusted up and down within a certain range to ensure the quality of the acquired data; the HIWIN six-degrees-of-freedom robot arm was fixed on the load table, and the XEG-32 two-finger electric gripper was mounted at the end and acted as the actuator for the robot grasping. This "eye-to-hand" approach [27] is adopted for calibrating the vision system according to the overall design plan, including the camera calibration and hand-eye calibration, where the specification of the calibration board is an 11 × 8 square checkerboard grid, the size of each square grid is 30 × 30 mm, and the accuracy is ±0.05 mm.

The camera calibration is based on the calibration toolbox of MATLAB 2020a to calibrate the depth camera of the Kinect V2. The corner points of the checkerboard grid can be detected according to the acquired calibration board image and the parameters of the calibration board, and the calibration is completed by returning the pixel point coordinates of the detected corner points. The final Kinect V2 depth camera internal reference is shown in Table 1.

**Figure 8:**
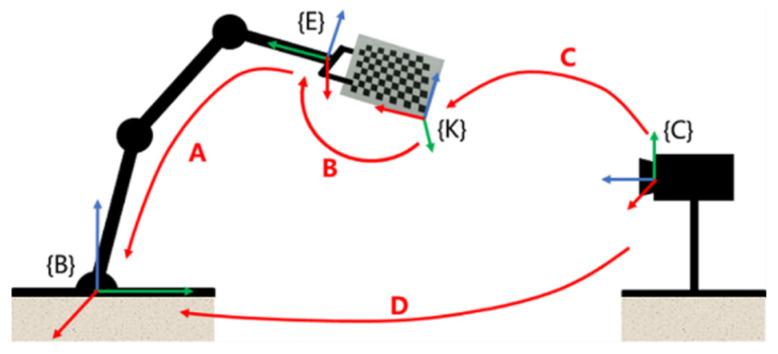
Schematic diagram of hand-eye calibration.

The purpose of hand-eye calibration is to solve the pose transformation matrix from the camera coordinate system to the manipulator coordinate system so as to convert the target yarn-bobbin poses from the camera coordinate system to the manipulator coordinate system. As shown in Figure 8, {B} is the base coordinate system of the manipulator; {E} is the coordinate system of the end of the manipulator; {K} is the calibration board coordinate system; {C} is the Kinect V2 depth camera coordinate system. **A** represents the pose of the end of the manipulator in the base coordinate system of the manipulator, which can be obtained by the positive solution of the robot kinematics; **B** represents the pose of the calibration board in the coordinate system of the end of the manipulator; **C** represents the pose of the calibration board in the camera coordinate system, that is, the external parameters in the camera calibration; **D** is the pose of the camera in the base coordinate system of the manipulator, that is, the final result of the hand-eye calibration.

Through the position conversion relationship, the process of the hand-eye calibration can be transformed into the solution **X** to **AX = XB**, that is, the conversion matrix from the Kinect V2 depth camera coordinate system {C} to the manipulator base coordinate system {B}, and the solution **X** is derived as follows, where the calibration accuracy is close to 99%.

(7) $$X=\left[\begin{array}{cccc} 0.0022 & 0.9999 & 0.0144 & 57.7571\\ -0.9999 & 0.0020 & 0.0109 & -24.0240\\ 0.0109 & -0.0144 & 0.9998 & -147.0713\\ 0 & 0 & 0 & 1.0000 \end{array}\right]$$

### 3.2. Grasping Experiments

**Figure 9:**
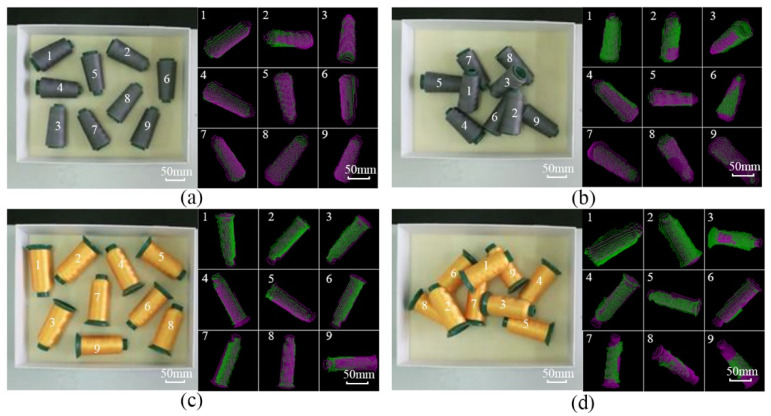
Experimental tests in different scenarios: (**a**) Tower yarn bobbin without contact scenario; (**b**) Tower yarn-bobbin stacking scenario; (**c**) Cylindrical yarn bobbin without contact scenario; (**d**) Cylindrical yarn-bobbin stacking scenario.

To evaluate the performance of the proposed robot visual recognition and grabbing method, field tests using two different yarn bobbin products with different specifications were conducted, as shown in Table 2. Two sets of scenarios (i.e., separated and disordered stacking) were designed for comparison and validation, and in each scenario, nine yarn bobbins were placed, as shown in Figure 9, with serial numbers 1 to 9.

**Figure 10:**
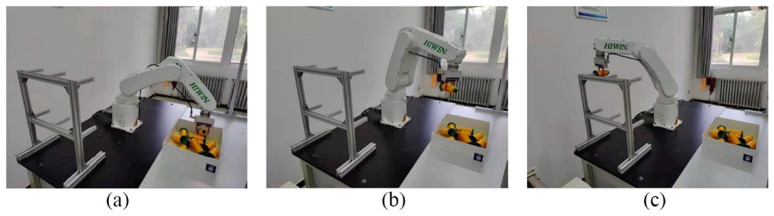
Diagram of the gripping process of part of the yarn bobbin. (**a**) grasping operation, (**b**) transmissing operation, and (**c**) loading operation.

In the two scenarios, each kind of yarn bobbin was tested for 150 grabs, and the box containing the yarn bobbins was placed in the working range of the robot, where the Kinect V2 camera took the yarn bobbin images and derived the poses of the yarn bobbins through the imaging process to determine the grabbing sequence according to the quantity of measured yarn-bobbin point clouds. Then, the yarn bobbin poses in the camera coordinate system were transferred to the hand-eye calibration; the robot-controlled gripper moved above the yarn bobbin and adjusted the end poses, grabbing the yarn bobbin, then moved the bobbins onto the rack. The grabbing process is shown in Figure 10.

The average time from the acquisition of target information by the vision system to the completion of grasping and grasping success rates are provided in Table 3.

The experimental results show that the proposed vision recognition and grasping system for the yarn bobbin robot can successfully grasp the yarn targets and complete the yarn loading work within the industrial time demand; the grasping success rate in the no-contact scenario is much higher compared with the disordered stacking scenario, and the operation time qass shorter in the no-contact scenario because there was interference between the yarn targets in the disordered stacking scenario. In addition, due to incomplete information, the robot failed to grab some bobbins, which led to a relatively low grasping success rate. However, one can note that the bobbin-loading robot is comparable to two workers in the traditional production line, as shown in Figure 1, and has a clear advantage over manual handling when working continuously for a long period of time. As a result, the work efficiency significantly improved, thus reducing the production costs and safety risks.

## 4. Conclusions

In this research, a new machine vision-based system is proposed for the yarn bobbin robots to complete the yarn-bobbin grabbing operation. An experimental platform was established to evaluate the proposed machine vision-based system. The main conclusions are as follows:

(1) By analyzing the yarn-bobbin loading processing, we develop a Kinect V2-based robot visual recognition and grabbing method, which erects a reality experimental platform for verification. Experimental results show that the robot is able to complete the grabbing operation within 10 seconds with a grabbing success rate of over 80%, which sufficiently meets the industrial requirements.

(2) By analyzing the yarn-bobbin point cloud data acquisition and pre-processing, the yarn-bobbin depth images acquired from the Kinect V2 camera can be converted into 3D point cloud data, and the noise points in the images are removed by pass-through filtering and MSAC fitting to yield the point cloud data of the yarn bobbins.

(3) By performing the rough registration and precise registration, the exact poses of the identified yarn bobbins can be extracted to help the robot correctly grab the yarn bobbins.

## Figures and Tables

**Table 1:** Kinect V2 depth camera calibration internal reference.

Name of Parameter	Data
Focal length	$\left[\begin{array}{cc} 359.2873 & 359.4936 \end{array}\right]$
Point coordinates	$\left[\begin{array}{cc} 252.6447 & 204.3739 \end{array}\right]$
Radial distortion	$\left[\begin{array}{cc} 0.0880 & -0.2112 \end{array}\right]$
Error	0.2374

**Table 2:** Yarn bobbin specifications.

Types	Diameter/mm		Height (mm)	Weight (kg)
**Tower-shaped**	Small 41	Big 52	112	0.11
Cylindrical	47		125	0.12

**Table 3:** Experimental results.

Experimental Scenarios	Experimental Subjects	Average Time Taken	Number of Experiments	Number of Successes	Success Rate
No contact	Tower-shaped	8.35 s	150	138	92.0%
	Cylindrical	8.07 s	150	139	92.7%
Unordered stacking	Tower-shaped	9.68 s	150	127	84.7%
	Cylindrical	9.82 s	150	129	86.0%
